# InsightSleepNet: the interpretable and uncertainty-aware deep learning network for sleep staging using continuous Photoplethysmography

**DOI:** 10.1186/s12911-024-02437-y

**Published:** 2024-02-14

**Authors:** Borum Nam, Beomjun Bark, Jeyeon Lee, In Young Kim

**Affiliations:** 1 Department of Electronic Engineering, Hanyang University, Seoul, Republic of Korea; 2 Department of Biomedical Engineering, Hanyang University, 222, Wangsimni-ro, Seoul, 04763 Republic of Korea

**Keywords:** Sleep staging, Artificial intelligence, Uncertainty, Interpretability, Photoplethysmography

## Abstract

**Background:**

This study was conducted to address the existing drawbacks of inconvenience and high costs associated with sleep monitoring. In this research, we performed sleep staging using continuous photoplethysmography (PPG) signals for sleep monitoring with wearable devices. Furthermore, our aim was to develop a more efficient sleep monitoring method by considering both the interpretability and uncertainty of the model’s prediction results, with the goal of providing support to medical professionals in their decision-making process.

**Method:**

The developed 4-class sleep staging model based on continuous PPG data incorporates several key components: a local attention module, an InceptionTime module, a time-distributed dense layer, a temporal convolutional network (TCN), and a 1D convolutional network (CNN). This model prioritizes both interpretability and uncertainty estimation in its prediction results. The local attention module is introduced to provide insights into the impact of each epoch within the continuous PPG data. It achieves this by leveraging the TCN structure. To quantify the uncertainty of prediction results and facilitate selective predictions, an energy score estimation is employed. By enhancing both the performance and interpretability of the model and taking into consideration the reliability of its predictions, we developed the InsightSleepNet for accurate sleep staging.

**Result:**

InsightSleepNet was evaluated using three distinct datasets: MESA, CFS, and CAP. Initially, we assessed the model’s classification performance both before and after applying an energy score threshold. We observed a significant improvement in the model’s performance with the implementation of the energy score threshold. On the MESA dataset, prior to applying the energy score threshold, the accuracy was 84.2% with a Cohen’s kappa of 0.742 and weighted F1 score of 0.842. After implementing the energy score threshold, the accuracy increased to a range of 84.8–86.1%, Cohen’s kappa values ranged from 0.75 to 0.78 and weighted F1 scores ranged from 0.848 to 0.861. In the case of the CFS dataset, we also noted enhanced performance. Before the application of the energy score threshold, the accuracy stood at 80.6% with a Cohen’s kappa of 0.72 and weighted F1 score of 0.808. After thresholding, the accuracy improved to a range of 81.9–85.6%, Cohen’s kappa values ranged from 0.74 to 0.79 and weighted F1 scores ranged from 0.821 to 0.857. Similarly, on the CAP dataset, the initial accuracy was 80.6%, accompanied by a Cohen’s kappa of 0.73 and weighted F1 score was 0.805. Following the application of the threshold, the accuracy increased to a range of 81.4–84.3%, Cohen’s kappa values ranged from 0.74 to 0.79 and weighted F1 scores ranged from 0.813 to 0.842. Additionally, by interpreting the model’s predictions, we obtained results indicating a correlation between the peak of the PPG signal and sleep stage classification.

**Conclusion:**

InsightSleepNet is a 4-class sleep staging model that utilizes continuous PPG data, serves the purpose of continuous sleep monitoring with wearable devices. Beyond its primary function, it might facilitate in-depth sleep analysis by medical professionals and empower them with interpretability for intervention-based predictions. This capability can also support well-informed clinical decision-making, providing valuable insights and serving as a reliable second opinion in medical settings.

## Background

Sleep is a state of reduced or absent consciousness in both mind and body, marked by relatively inactive sensory organs, minimal muscle movement, and decreased responsiveness to stimuli [1]. According to the American Academy of Sleep Medicine (AASM), sleep consists of four distinct stages [2]. These stages can be categorized into two main phases: Rapid Eye Movement (REM) sleep, characterized by rapid eye movements, and non-REM sleep. Non-REM sleep is further divided into three stages, known as N1, N2, and N3, each defined by specific electroencephalogram (EEG) patterns following established sleep stage evaluation guidelines. In a typical sleep cycle, REM sleep follows non-REM sleep, and this non-REM-REM cycle repeats throughout the night. Individuals experiencing sleep disorders or poor sleep quality may encounter difficulties with daytime concentration, memory impairment, and persistent fatigue. The quality of sleep is closely linked to these sleep stages, with reduced REM sleep and N2 stages often resulting in diminished sleep quality [3, 4]. Therefore, monitoring sleep becomes crucial for identifying and addressing issues related to poor sleep quality, which can lead to various disadvantages. The polysomnography primarily involves the use of electroencephalography (EEG), electrooculography (EOG), and electromyography (EMG). Additionally, it may include electrocardiogram (ECG) monitoring, video recording, airflow measurement, oxygen saturation monitoring, abdominal movement tracking, and audio recording [5]. Polysomnography is used to facilitate the diagnosis of sleep disorders and the assessment of sleep quality. However, it requires overnight stays at a hospital or sleep center, potentially leading to the first-night effect. This phenomenon may lead to a decrease in sleep efficiency, frequent awakenings, and an increased proportion of light sleep, making it challenging to accurately assess sleep quality [6]. Additionally, polysomnography is resource-intensive, requiring the attachment of multiple sensors, which can be uncomfortable and significantly expensive [7]. To overcome these limitations, there is a growing demand for sleep monitoring methods that utilize wearable devices. Using wearable devices enables sleep monitoring in the comfort of one’s home, mitigating the first-night effect while reducing costs. Consequently, our research aims to develop an automated sleep staging algorithm for wearable sensor-based sleep monitoring, offering these advantages. Wearable devices include various modalities, such as electrocardiography (ECG), accelerometry, audio, and electrodermal activity (EDA). Numerous sleep staging algorithms were developed based on the signals that can be acquired from these wearable devices [8]. In this study, we focus on developing a sleep staging algorithm based on photoplethysmography (PPG) signal. PPG signal is optical measurements used for heart rate monitoring and detecting changes in blood volume. PPG sensor is commonly found in watch-like devices and is adaptable to various other wearable devices, making it a convenient choice for sleep staging. Importantly, there is a strong connection between sleep stages and the autonomic nervous system, as both sleep and the autonomic nervous system are regulated by the same central nervous system mechanisms [9, 10]. Furthermore, there is evidence supporting changes in heart rate, respiratory rate and blood pressure with each stage of the sleep cycle [11–14]. Consequently, since the autonomic nervous system regulates the cardiovascular and respiratory systems, PPG can serve as a reliable proxy for sleep staging.

These advantages of PPG have led to numerous sleep staging studies that employ PPG. With the advancement of deep learning algorithms, recent PPG-based sleep staging algorithms use two primary methods. The first method involves creating a pre-trained model using other signals, such as ECG, to compensate for the lack of PPG datasets, followed by transfer learning based on the model. In a previous study [15], a long short-term memory (LSTM) model that utilizes heart rate variability was fine-tuned to classify four classes (wake, light sleep, deep sleep, and REM sleep) using a PPG dataset. Another prior study [16] utilized an ECG dataset to train an LSTM that classified the same four classes and fine-tuned it with the PPG dataset. The second method is to train and develop a classification model using the PPG dataset alone, without transfer learning. In [17], an algorithm for classifying four classes was developed using a model that combines convolutional neural network (CNN) and bidirectional gated recurrent unit (GRU). In [18], a model combining CNN and LSTM was developed, and its performance in classifying three classes (wake, non-REM sleep, REM sleep), four classes, and five classes (wake, N1, N2, N3, REM sleep) was evaluated. Among these, SleepPPG-Net [19], a state-of-the-art (SOTA) algorithm, utilized time-distributed residual convolution (ResConv) blocks and temporal convolutional network (TCN), and verified its performance in classifying four classes using two public datasets. This study compared deep learning-based models using continuous PPG signals with conventional derived time series and feature engineering methods, concluded that SleepPPG-Net, which uses PPG signals without a feature extraction process, exhibited the best performance. Since automatic sleep staging requires the utilization of complex information from very large datasets, existing feature extraction methods may be influenced by several factors [5]. Building on this, our study aimed to develop a 4-class sleep staging algorithm using continuous PPG data based on deep learning. In particular, our goal was to create an algorithm that could deliver performance without relying on transfer learning using other signals like ECG, making it useful in cases where transfer learning was not feasible (e.g., when utilizing only PPG collected by wearable devices).

While existing PPG-based sleep staging algorithms yielded impressive results, they struggled due to a lack of interpretability and the misclassification of light sleep as deep or REM sleep. Interpretability is crucial in the biomedical field, as it ensures patient safety, reliability, and supports medical decision-making by healthcare providers [20]. Similarly, in the case of sleep staging, analyzing data collected at home by wearable devices and presenting the results to clinicians can have the advantage of improving and optimizing care by supporting medical decisions, provided that the algorithms used in the analysis are interpretable. As mentioned in the studies that developed automated sleep scoring algorithms using PSG data, it is crucial in sleep staging to develop algorithms that are, above all, reliable and stable [5, 21]. While some studies focused on these benefits and interpreted sleep stage prediction results using different signals [22–24], there is a scarcity of algorithms that utilize PPG to ensure both performance and interpretability. Moreover, introducing a technique to measure prediction uncertainty in cases where confusion arises among specific sleep stage classes could enhance human decision-making by providing information about the degree of uncertainty in the predictions and allowing for decisions to be postponed [25, 26]. This approach is similar to studies that have measured uncertainty in other biomedical domains and can enable synergies between humans and AI [27–29]. Existing methods for measuring uncertainty in the predictions of deep learning models include computing uncertainty over the trained model, such as normalized entropy [30] and softmax response [31], and computing uncertainty using a dropout layer embedded in the model, such as Monte-Carlo dropout [32]. Among these conventional methods, newer approaches with enhanced performance were developed. One of these methods [33, 34] measures uncertainty by employing selective prediction using a rejection option [33], which is improved by incorporating a separate rejection component into the model structure. However, this outstanding method requires simultaneous optimization of both the classification and rejection components, potentially leading to a lengthy optimization process, especially for complex data such as medical data. In addition to this approach, the most recent research focuses on utilizing an energy score to assess the uncertainty of model prediction results [34]. This technique addresses the issue of overconfidence observed in traditional methods, and prediction uncertainty can be readily obtained by calculating the energy score during the inference stage without requiring any additional modifications to the model. Thanks to its versatility, energy scoring provides flexibility and can be employed with various model architectures and datasets, establishing it as a widely adopted method for uncertainty estimation.

In this study, we aimed to develop a sleep staging model that possesses a better understanding of classification and can maximize synergy between medical professionals and AI. To achieve this, we accomplished this by enhancing the interpretability of the sleep staging algorithm through the use of continuous PPG data based on deep learning and by incorporating a measure of prediction result uncertainty.

## Methods & Materials

### InsightSleepNet

**Fig. 1:**
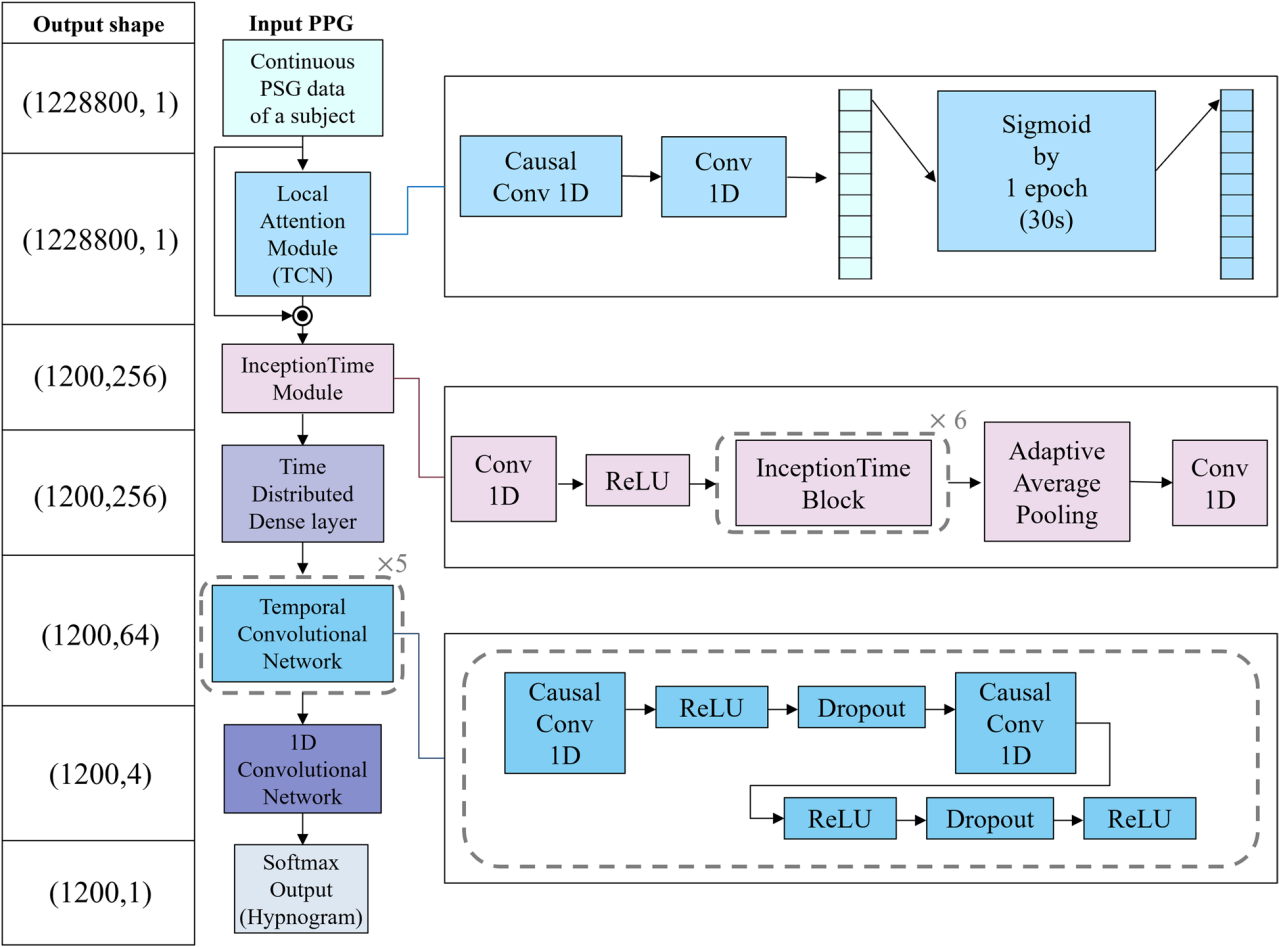
InsightSleepNet architecture This was done to discern which segment of the PPG signal influenced the prediction for each epoch in continuous PPG. Additionally, to identify light sleep, we incorporated the ‘3-minute rule’ [35], an established sleep staging technique that analyzes data over 3-minute intervals, into our local attention module. To achieve this, we set the kernel size of the causal convolutional layer, the initial layer of the local attention module, to 7168 (equivalent to 7 epochs) and a stride of 1. This configuration allowed the layer to cover the preceding 3 minutes of the epoch to be predicted. The causal convolutional layer of the TCN model has the characteristic of applying zero padding equal to ‘kernel size - 1’ on both sides of the input data sequence. This feature enabled us to perform computations as intended from the very first epoch. After the causal convolution, we added a 1D convolutional layer with a kernel size of 1 and a stride of 1 to generate an output with a size of 1. Following that, we applied a sigmoid operation every 1024 epochs, corresponding to the PPG signal length within a single epoch. This enabled the calculation of an attention score for each epoch across the entire PPG sequence, considering causality and the influence of the previous 7 epochs. Consequently, an attention score ranging from 0 to 1 was calculated for each epoch

Our goal was to develop a 4-class sleep staging model based on continuous PPG data, with a focus on interpretability and the ability to estimate prediction uncertainty. To accomplish this objective, we created InsightSleepNet, whose overall structure is depicted in Fig. 1. InsightSleepNet contains five main components: a local attention module, an InceptionTime module, a time-distributed dense layer, a temporal convolutional network (TCN), and a 1D convolutional network (CNN).

To develop a model with interpretability, we introduced a local attention module into the model structure in this study. To design this local attention module, we drew inspiration from a structure called TCN [36]. TCN presents several advantages, such as the employment of causal convolutions that consider the temporal causality of time series data, dilated convolutions with an expansive receptive field capable of representing a wide spectrum of time series data, and the incorporation of residual connections that contribute to the stability of training for TCN models with multiple layers. In our research, we aimed to construct a local attention module to gain insight into the interpretation of prediction results for each epoch (30 seconds), which serves as the fundamental unit for sleep stage classification.

**Fig. 2:**
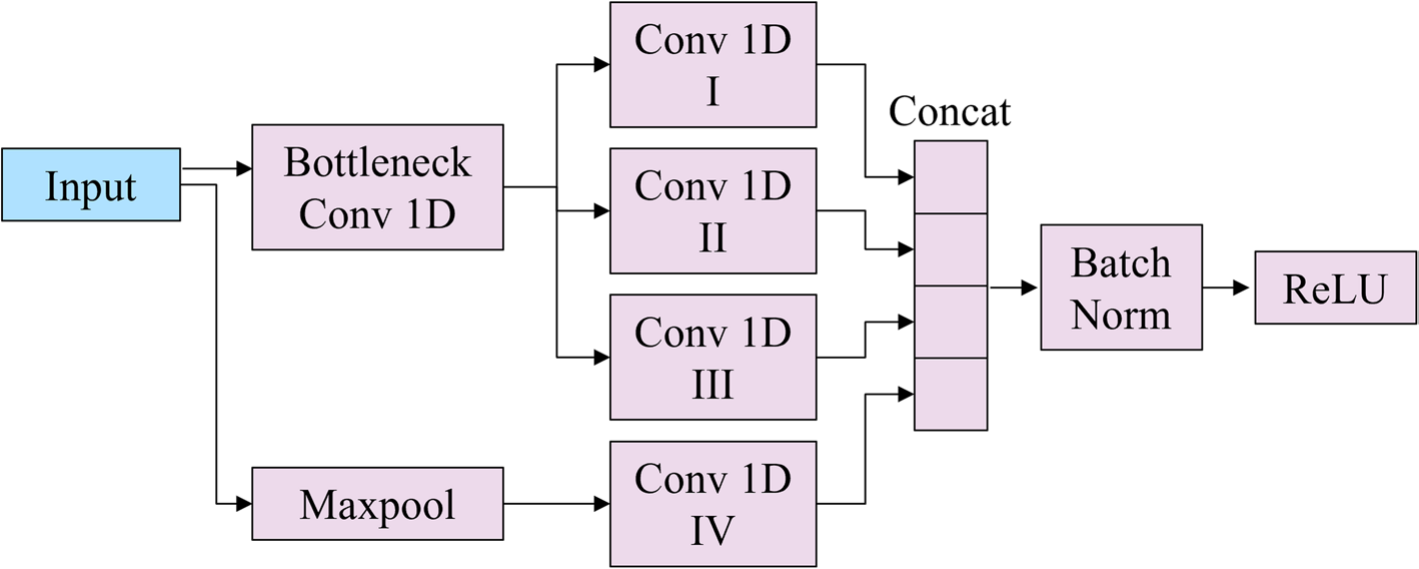
Inception time block structure

After the local attention module, we designed a structure capable of effectively compressing the weighted input and learning its features. For this purpose, we utilized the InceptionTime structure [37]. InceptionTime has a bottleneck architecture, various convolutional operations with different time scales, and includes residual connections, making it well-suited for learning long time series data. Due to these structural elements, its performance outperformed that of existing models designed for time series classification, leading us to adopt this structure. The InceptionTime module in the InsightSleepNet developed in this study is depicted in Fig. 1. The InceptionTime module in InsightSleepNet has a 1D convolutional layer with a channel size of 32, a stride of 20, and a kernel size of 40, followed by ReLU activation. It subsequently incorporates 6 InceptionTime blocks. The structure of the InceptionTime block is illustrated in Fig. 2. The kernel sizes of the three convolutional layers present in the InceptionTime block (Conv I, Conv II, Conv III in Fig. 2) are 5, 11, and 23, respectively, with a stride of 1. The convolutional layer following max-pooling (Conv IV in Fig. 2) has a kernel size of 1 and a stride of 1. In InsightSleepNet, a total of 6 InceptionTime blocks are employed. As a result, the channel sizes for these blocks are set to 32, 32, 64, 64, 128, 256, while the filter sizes are 8, 16, 16, 32, 64, 128, and the bottleneck channel sizes are 8, 16, 16, 16, 32, 32. The output of the InceptionTime block is subsequently obtained as the final output of the InceptionTime module after passing through an adaptive average pooling layer, followed by a 1D convolutional layer with a kernel size of 1.

Furthermore, for achieving optimal classification performance, we devised a layer structure inspired by a hybrid architecture, combining the time-distributed dense layer and TCN, as employed in state-of-the-art (SOTA) technology [19]. The output of the InceptionTime block served as the input. We utilized the time-distributed dense layer to produce outputs for each time step. Subsequently, we compressed the final features using five temporal blocks of TCN and a 1D convolutional network to generate the ultimate output. The temporal block structure of TCN, as illustrated in Fig. 1, includes the following elements: an initial 1D causal convolutional layer, ReLU activation, dropout layer, secondary 1D causal convolutional layer, ReLU activation, and another dropout layer. Across all five temporal blocks, the channel sizes remained consistent at 64, with fixed kernel sizes and strides of 8 and 1, respectively. A uniform dropout ratio of 0.2 was applied, and dilation sizes for each temporal block were set to 1, 2, 4, 8, and 16. The 1D convolutional layer responsible for generating the final output had a kernel size of 1 and a stride of 1. The shape of the final output was (1200, 4), representing values for four classes (wake, light sleep, deep sleep, REM) across 1200 epochs. The ultimate prediction was calculated using softmax.

### Uncertainty measure

To estimate the uncertainty of the results predicted by the designed model, this study utilized a method for measuring the energy score [34]. This technique is employed to detect whether inputs are out-of-distribution (OOD), measuring the uncertainty that arises when the model encounters inputs differing in patterns from the training data. Consequently, it enables the detection of cases where the model exhibits low confidence in predictions, particularly for samples that do not align with the training set. When a neural classifier, represented as *f*(*x*): *ℝ*^*D*^ → *ℝ*^*K*^, maps an input *x* ∈*ℝ*^*D*^ to logits in the form of K real-value numbers, the energy-based model (EBM) [38] employs these logits to generate a categorical distribution using the softmax function as follows:

1 $$p\left(y|x\right)=\frac{e^{f_y(x)/T}}{\sum_i^K{e}^{f_i(x)/T}}$$

where *T* is the temperature parameter derived from the Gibbs distribution, and *f*_*y*_(*x*) represents the *y*^th^ index of *f*(*x*), the logit corresponds to the *y*^th^ class label. Therefore, for a given input (*x*, *y*) the Helmholtz free energy *E*(*x*) can be expressed as the free energy function *E*(*x*; *f*) as follows, based on eq. (1), in terms of the denominator of the softmax activation:

2 $$E\left(x;f\right)=-T\cdot \log \sum_i^K{e}^{f_i(x)/T}$$

**Fig. 3:**
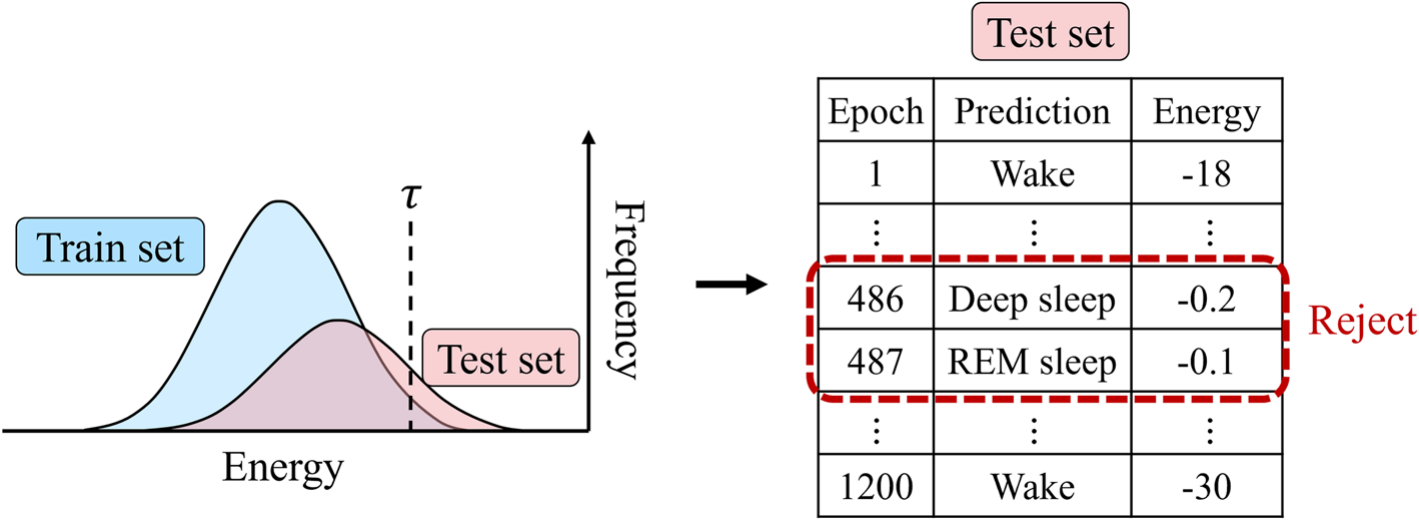
Energy score thresholding

As a result, the energy score is a value computed for a given input *x*, utilizing the probability density of the inputs. According to the theory of the EBM model, it yields low values for observed data and relatively high values for unobserved data. Equation (2) is employed for selective prediction of classification results, and the selector G used in this process is expressed as follows:

3 $$G\left(x;\tau; f\right)=\left\{\begin{array}{c}f(x),\kern0.5em if\ E\left(x;f\right)\le \tau \\ {} do{n}^{\prime }t\ know\ (rejection),\kern0.5em if\ E\left(x;f\right)>\tau\ \end{array}\right.$$

where *τ* represents the energy threshold. To make the selector *G* practically effective, the threshold must be determined empirically. In this study, we set the test set’s threshold by utilizing the energy score distribution of the training set. Previous studies [5, 15–19] noted that existing sleep staging algorithms often misclassify deep sleep and REM sleep stages as light sleep, despite effectively classifying the wake class. We considered this misclassification to have contributed to the decline in the performance of sleep staging results. To address this, during inference time, when an input sample’s energy score exceeds the threshold, we opted to withhold judgment and reject the prediction, as illustrated in Fig. 3.

### Datasets

In this study, we used a total of three polysomnography datasets, all of which were publicly available databases: MESA [39, 40], CFS [39, 41], and CAP [42]. For the MESA dataset (Multi-Ethnic Study of Atherosclerosis), we used data from 2054 subjects, which included PPG signals sampled at 256 Hz. Following previous research [19], we developed and evaluated our model by splitting the dataset into a training set with 1850 subjects and a test set with 204 subjects using a hold-out strategy. For the CFS dataset (Cleveland Family Study), we utilized data obtained from 320 subjects with a PPG sampling rate of 256 Hz. For model evaluation, we employed a 4-fold cross-validation approach based on previous research [19]. For the CAP dataset (Cyclic Alternating Pattern), sourced from PhysioNet [43], we used the PPG signal data from a total of 24 subjects with sleep disorders (insomnia:5, nocturnal frontal lobe epilepsy:8, REM behavior disorder:7, no pathology:4). The PPG signals in this dataset were sampled at 128 Hz. We validated overall performance using 4-fold cross-validation (training set: 18, validation set: 6). All three datasets used in this study were annotated into six classes based on the Rechtschaffen & Kales manual by expert annotators. Referring to prior research [19], datasets are described in more detail in Table 1. This research received ethical approval from the Institutional Review Board of Hanyang University (#HYUIRB-202309-009), and the requirement for informed consent was waived by the institution. All procedures were conducted in accordance with relevant guidelines and regulations.

**Table 1:** PSG database overview with statistics is presented using the median and inter quartile range (IQR)

	MESA	CFS	CAP
Patients	2054	320	24
Gender (M:F)	1:1.2	1:1.2	1:1
Total windows	2.35 M	0.37 M	0.03 M
Duration (hrs)	10 [9–10]	10 [9–10]	9 [8–10]
Age (yrs)	68 [62–76]	42 [21–54]	44 [29–71]
Wake (%)	37 [30–47]	34 [27–44]	14 [8–30]
Light (%)	43 [36–50]	39 [29–46]	39 [31–45]
Deep (%)	5 [1–10]	12 [7–19]	25 [20–30]
REM (%)	11 [7–14]	11 [8–14]	15 [13–22]

### Preprocessing

We performed signal processing to use continuous PPG samples from the database as inputs for the model. The preprocessing was conducted with reference to a previous study [19] for fair comparison. We utilized an 8th-order zero-phase low-pass Chebyshev Type II filter with a cutoff frequency of 8 Hz and a stop-band attenuation of 40 dB for signal filtering. After this, we applied a 10th order polynomial for detrending the signal. As the range of PPG signals varied across each dataset, we applied min-max normalization to ensure that the model could interpret PPG signal variations and scale them to a range between 0 and 1, enabling sleep staging. In order to have each epoch comprising 1024 samples, we sampled the PPG signal at a frequency of 34.3 Hz. To prepare the continuous PPG signals for the input layer of the deep learning model, we standardized the total length of the PPG signal to 10 hours, equivalent to 1200 epochs. Any samples longer than this length were truncated, and those shorter were zero-padded. The zero-padded epochs were not used for loss calculation and performance evaluation. Since all three datasets were labeled according to the R&K manual, we transformed the sleep staging into a 4-class label format. The S1 and S2 stages were grouped together as the ‘light sleep’ class, while the S3 and S4 stages were consolidated into the ‘deep sleep’ class. The wake and REM stages were retained unchanged.

### Model setting

In this study, InsightSleepNet with 1,922,397 trainable parameters was trained with a batch size of 2 and for 100 epochs. It was optimized using the RMSprop optimizer with learning rates of 0.001 for MESA. The Adam optimizer was used with learning rates of 0.001 for CFS and 0.001 for the CAP dataset. Because the MESA dataset was the largest, we performed a transfer learning on the CFS and CAP datasets using the entire pre-trained InsightSleepNet model, which was initially trained on the MESA dataset. During the transfer learning, the weights were not frozen. Due to different class distributions in the datasets used in this study, weights were assigned to each class sample based on the training set, and the model was trained using the Negative Log Likelihood (NLL) loss function. We empirically selected the hyperparameters and the optimizer used for training through repeated testing with a grid search method. Due to the relatively long length of each continuous PPG sample, the maximum batch size our computing resources could handle was limited to 2. Similarly, through the grid search, we confirmed that a batch size of 2 achieved the best performance across all datasets. Our model was designed using the PyTorch framework and trained using an AMD Ryzen 55,600X 6-Core Processor, an NVIDIA GeForce RTX 3090 GPU, and 64.0GB of RAM.

### Model performance evaluation

For the evaluation of InsightSleepNet, we presented accuracy and Cohen’s kappa coefficient based on previous studies. Accuracy in (Eq. 4) represents the proportion of correct predictions among all the samples, while Cohen’s kappa in (Eq. 5) quantifies the level of agreement between experts. Additionally, we used the weighted F1 score to evaluate performance, considering the imbalanced class distribution, which was not addressed in previous studies [19, 44]. The F1 score in (Eq. 6) represents a balance between precision and recall. Using this, we calculated the weighted F1 score to account for the imbalance between classes. In the equations below, TP stands for True Positive, TN for True Negative, FN for False Negative, and FP for False Positive. Additionally, *Pr*(*a*) represents the probability of agreement between the evaluations of two assessors, and *Pr*(*e*) represents the probability of chance agreement between the two assessors. These metrics were computed for predictions on each epoch of the entire continuous PPG signal dataset.

4 $$Accuracy=\frac{TP+ TN}{TP+ FP+ FN+ TN}$$

5 $$Cohe{n}^{\prime }s\ kappa=\frac{\mathit{\Pr}(a)-\mathit{\Pr}(e)}{1-\mathit{\Pr}(e)}$$

6 $$F1\ score=\frac{2 TP}{2 TP+ FP+ FN}$$

To ensure dependable classification in this study, we adopted a classification approach based on an energy score threshold. For samples that did not meet the threshold criteria, we considered them to have insufficient confidence and rejected them. We examined the distribution of energy scores in the training set to empirically choose energy score threshold. In this study, we defined energy score thresholds based on the energy score distribution using four criteria: the top 80%, top 85%, top 90%, and top 95%. These energy score thresholds were used to determine rejection during validation, and we evaluated the resulting performance improvements. Additionally, to validate whether the developed model could be utilized as a tool for evaluating sleep quality, we presented the results of estimating sleep parameters in this study. The presented sleep parameters include four variables: total sleep time (TST) and sleep-stage fractions (*FR*_*Light*_, *FR*_*Deep*_, *FR*_*REM*_), as represented by Eqs. (7) and (8). We assessed the validity of InsightSleepNet by comparing the estimated sleep parameters with the ground truth sleep parameters for each subject, using correlation analysis. Similarly to the calculation of loss and performance, the zero-padded and truncated parts were not utilized in the estimation of the sleep parameters. For the samples shorter than 10 hours, the sleep parameters were estimated based on their original number of epochs. For the recordings exceeding 10 hours, the initial 1200 epochs were used for the sleep parameter estimation. When evaluating the results of applying the energy score threshold, we excluded rejected samples from the overall evaluation.

6 $$TST=\sum Light\ sleep+\sum Deep\ sleep+\sum REM\ sleep\ (minute)$$

7 $${FR}_{Stage}=\frac{\sum Stage}{TST}\times 100\ \left(\%\right)$$

## Result

### Classification performance

In this study, we evaluated the performance of InsightSleepNet for PPG-based sleep stage classification using three datasets: MESA, CFS, and CAP. As results, we presented the classification performance for each dataset, along with the performance improvements achieved through energy-based rejection. Additionally, we examined the rejection rates for each stage using energy-based rejection and presented the results for sleep parameter prediction as well as model interpretation. In this section, we conducted a comparative analysis of classification performance before and after applying the energy score threshold for each dataset and compared it with the results from previous studies. Additionally, we compared our performance with the SOTA techniques for sleep staging using PPG from the same datasets. As mentioned earlier, we set the energy score thresholds based on the top 80, 85, 90, and 95% of the training set, presenting numerical results for various metrics. Firstly, we assessed the performance on the MESA dataset, which consisted of 204 test subjects selected randomly. We followed the same hold-out validation approach as previous research [19]. The InsightSleepNet without thresholding in this study achieved a classification accuracy of 84.2% and a Cohen’s kappa (κ) of 0.742. We obtained a weighted F1 score of 0.842 (for each class, Wake: 0.894, Light Sleep: 0.845, Deep Sleep: 0.330, REM Sleep: 0.770). When comparing these metrics to those reported for the SleepPPG-net, BM-FE, and BM-DTS models in previous research [19], our model either outperformed them or showed similar performance across the provided metrics. Subsequently, we applied energy score thresholding to InsightSleepNet to reject samples with low confidence. As a result, by employing four different thresholds, we observed accuracies ranging from 84.8 to 86.1%, κ values varying from 0.752 to 0.777, and weighted F1 scores ranging from 0.848 to 0.861. Lowering the energy score threshold resulted in more rejections and improved performance, enabling more confident sample classification (Table 2).

**Table 2:** Performance evaluation of the model on the MESA test dataset (*n* = 204) and a comparison before and after energy score thresholding

	InsightSleepNet (without thresholding)	0.80 Energy threshold	0.85 Energy threshold	0.90 Energy threshold	0.95 Energy threshold	BM-FE [19]	BM-DTS [19]	Sleep PPG-Net [19]
Accuracy	0.842	0.861	0.857	0.853	0.848	0.78	0.76	0.83
Cohen’s kappa (κ)	0.742	0.777	0.769	0.761	0.752	0.66	0.64	0.74
Weighted F1 score	0.8420	0.8613	0.8572	0.8528	0.8479	–	–	–

The ‘-’ symbol indicates that the corresponding metric is not provided in the study

We applied InsightSleepNet to the CFS dataset and conducted a 4-fold validation on 320 subjects, following the methodology outlined in prior research [19]. Each fold involved a distinct test set comprising 80 subjects without overlaps, resulting in a total of 320 validation instances. We consistently applied energy score thresholding and evaluation metrics, following the same approach used for the MESA dataset. Before applying energy score thresholding, InsightSleepNet demonstrated noteworthy performance with an accuracy of 80.6%, a Cohen’s kappa (κ) of 0.718 and a weighted F1 score of 0.808. These results outperformed models from previous studies (accuracy: 63–76%, κ: 0.47–0.67). In alignment with our approach for the MESA dataset, we applied energy score thresholding using four distinct criteria. As a result, for each of these criteria, we observed an enhanced performance, with accuracies ranging from 81.9 to 85.6%, κ values ranging from 0.738 to 0.793 and weighted F1 scores varying from 0.821 to 0.857. These outcomes are summarized in Table 3.

**Table 3:** Performance evaluation of the model on the CFS dataset (*n* = 320) and a comparison before and after energy score thresholding

	InsightSleepNet (without thresholding)	0.80 Energy threshold	0.85 Energy threshold	0.90 Energy threshold	0.95 Energy threshold	BM-FE [19]	BM-DTS [19]	Sleep PPG-Net [19]
Accuracy	0.806	0.856	0.844	0.832	0.819	0.63	0.69	0.76
Cohen’s kappa (κ)	0.718	0.793	0.777	0.758	0.738	0.47	0.53	0.67
Weighted F1 score	0.8082	0.8574	0.8461	0.8337	0.8210	–	–	–

The ‘-’ symbol indicates that the corresponding metric is not provided in the study

Finally, our model was applied to the CAP dataset, which consists of sleep study data from 24 subjects. Our model evaluated through 4-fold cross validation. We ensured that each fold had a distinct test set, avoiding overlaps. This resulted in a total of 4 validation sets (n = 24). Without the energy score thresholding, InsightSleepNet showed an impressive performance, achieving an accuracy of 80.6%, a Cohen’s kappa (κ) of 0.730 and a weighted F1 score of 0.805. These results already outperformed previous research [44], which focused on a four-class model. Subsequently, we applied four distinct energy score thresholds, following the same procedure as with the previous datasets. As seen in our previous results, reducing the energy score threshold led to higher rejection ratios while enhancing the overall model performance. The summary of these results is presented in Table 4.

**Table 4:** Performance evaluation of the model on the CAP dataset (*n* = 24) and a comparison before and after energy score thresholding

	InsightSleepNet (without thresholding)	0.80 Energy threshold	0.85 Energy threshold	0.90 Energy threshold	0.95 Energy threshold	Zhao et al., 2021 [44]
Accuracy	0.806	0.843	0.835	0.825	0.843	0.77
Cohen’s kappa (κ)	0.730	0.786	0.775	0.760	0.786	0.69
Weighted F1 score	0.8046	0.8417	0.8342	0.8242	0.8133	–

The ‘-’ symbol indicates that the corresponding metric is not provided in the study

### Energy based rejection

**Fig. 4:**
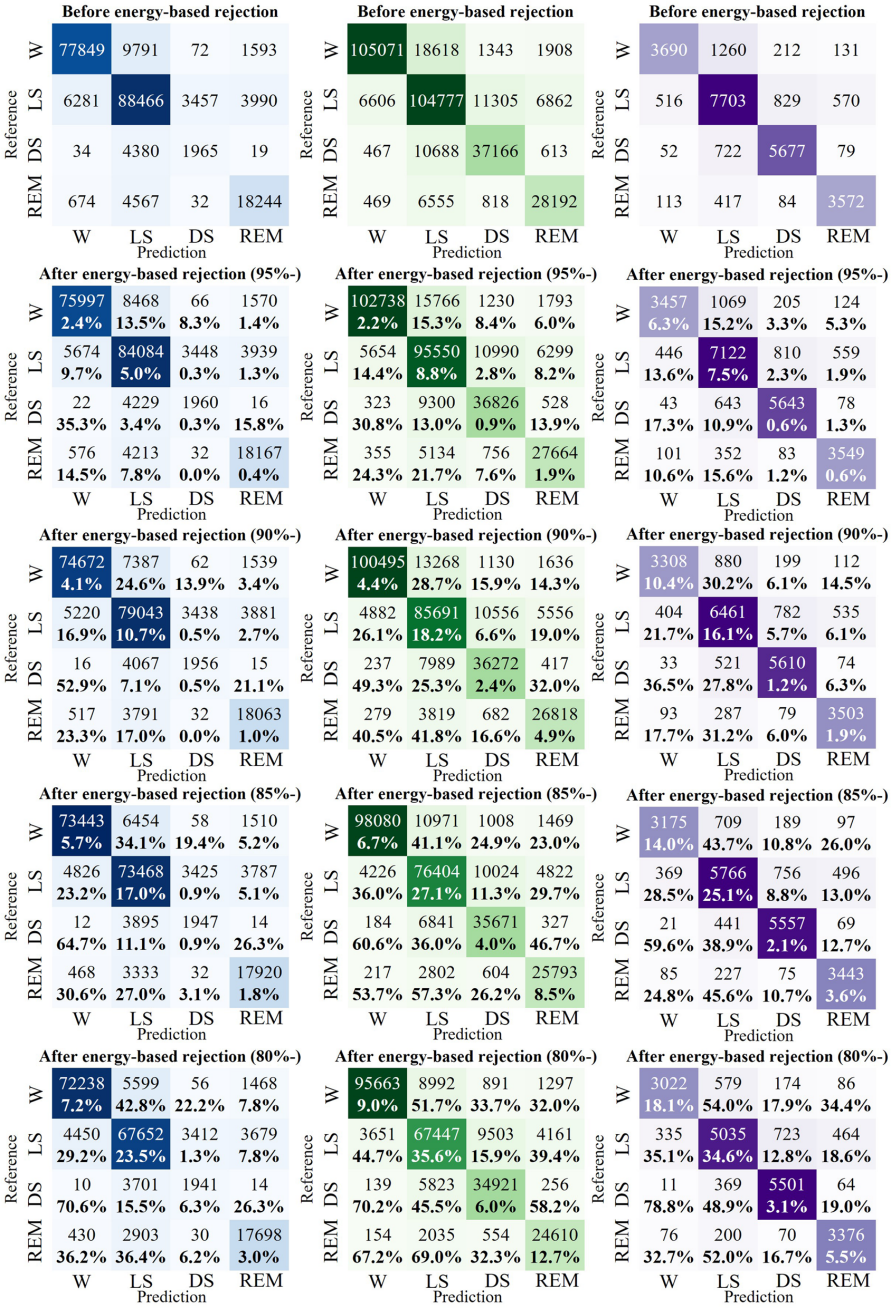
Proportion of samples rejected based on four different energy score thresholds for each dataset. The rejection rate is calculated with respect to the state before energy-based rejection. The blue confusion matrix represents the MESA dataset, the green one corresponds to the CFS dataset, and the purple one represents the CAP dataset. LS means 'Light sleep' and DS means 'Deep sleep'. W means 'Wake'

The utilization of energy score-based thresholding as a rejection technique in this study led to performance improvements. In this section, we examined changes in the rejection ratios for each sleep stage class. Using the top 90% of the energy score distribution from the training set as a threshold, we observed a rejection rate of 4% for samples classified as ‘wake’ in the MESA dataset that were actually ‘wake’ samples. Among the samples classified as ‘wake’, those whose actual classes were ‘light sleep’, ‘deep sleep’, and ‘REM sleep’ were rejected at rates of 16.9, 52.9, and 23.3%, respectively. Therefore, relatively uncertain samples tended to have a higher rejection rate compared to correctly classified samples. This trend was consistently observed across all three datasets and became more pronounced as we narrowed the tolerance range. This trend became more pronounced as we lowered the threshold. We have summarized these findings in Fig. 4.

### Estimate sleep parameters

**Fig. 5:**
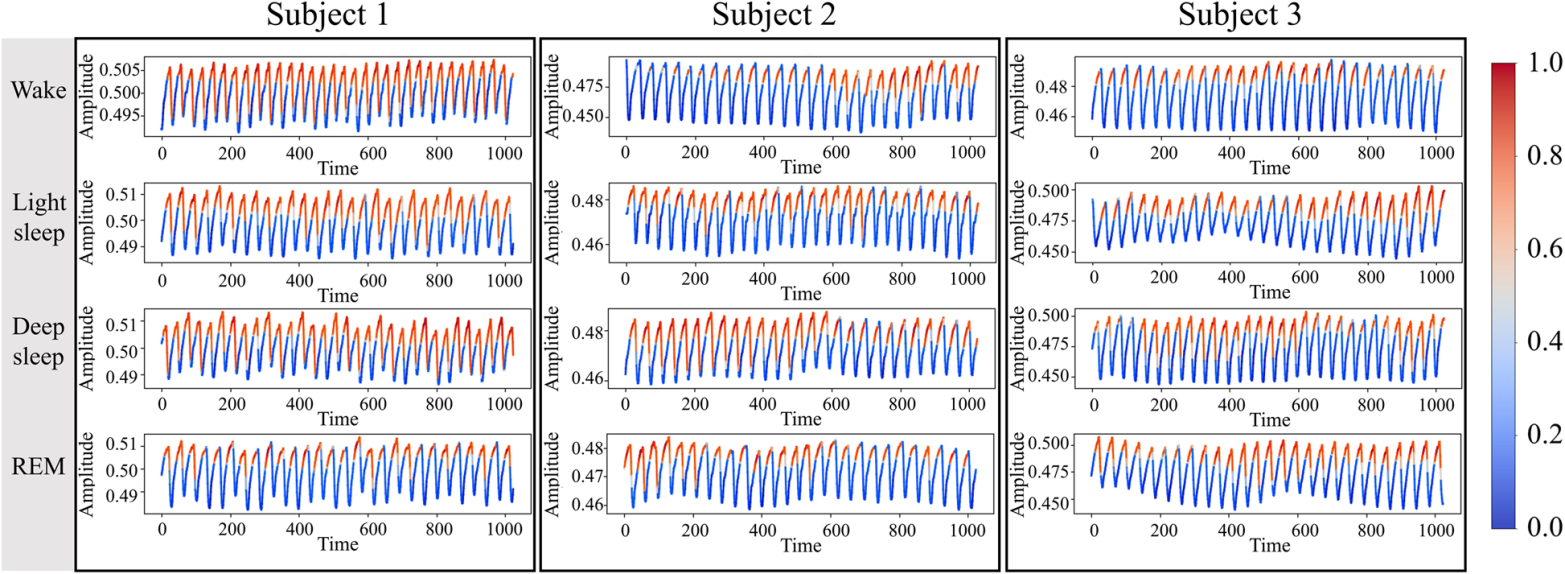
Estimation of sleep parameters in the CFS dataset (n = 320) before and after applying the energy score threshold. The red line represents before applying the energy score threshold, and the blue line represents after applying the energy score threshold

In this section, we aimed to validate whether InsightSleepNet can effectively estimate actual sleep quality. We estimated four sleep parameters: Total Sleep Time (TST), Light fraction, Deep fraction, and REM fraction, and compared them with the ground truth. This evaluation was conducted using three datasets, with the CFS dataset used for visualization purposes (Fig. 5). We assessed the reliability of confidence-based classification by examining the correlation coefficients between the estimated parameters from each subject’s data and the ground truth parameters, both before and after applying the energy score threshold. For the MESA dataset, before applying the energy score threshold, InsightSleepNet achieved the following correlation coefficients: *r* of TST was 0.969, *r* of Light fraction was 0.671, *r* of Deep fraction was 0.270, and *r* of REM fraction was 0.809. However, when an energy score threshold of 0.80 was applied, there were noticeable improvements in all sleep parameters. Furthermore, when we applied the model to the CFS and CAP datasets, we obtained the following correlation coefficients for TST: *r* of 0.913 for CFS and *r* of 0.810 for CAP, Light fraction: *r* of 0.796 for CFS and *r* of 0.827 for CAP, Deep fraction: *r* of 0.889 for CFS and *r* of 0.936 for CAP, and REM fraction: *r* of 0.781 for CFS and *r* of 0.790 for CAP. The application of energy score thresholding resulted in an average improvement of 0.054 in the correlation coefficients for CFS and 0.068 for CAP in the model performance for each dataset. A summary of these results is provided in Tables 5 and 6.

**Table 5:** The correlation coefficients between estimated sleep parameters and ground truth before energy score thresholding for each dataset

	MESA	CFS	CAP
TST	*r* = 0.969, *r*^2^ = 0.938	*r* = 0.913, *r*^2^ = 0.834	*r* = 0.810, *r*^2^ = 0.654
Light sleep fraction	*r* = 0.671, *r*^2^ = 0.450	*r* = 0.796, *r*^2^ = 0.768	*r* = 0.827, *r*^2^ = 0.684
Deep sleep fraction	*r* = 0.270, *r*^2^ = 0.073	*r* = 0.889, *r*^2^ = 0.790	*r* = 0.936, *r*^2^ = 0.877
REM sleep fraction	*r* = 0.809, *r*^2^ = 0.573	*r* = 0.781, *r*^2^ = 0.611	*r* = 0.790, *r*^2^ = 0.624

**Table 6:** The correlation coefficients between estimated sleep parameters and ground truth after applying the energy score threshold (0.80) for each dataset

	MESA	CFS	CAP
TST	*r* = 0.981, *r*^2^ = 0.964	*r* = 0.938, *r*^2^ = 0.880	*r* = 0.889, *r*^2^ = 0.791
Light sleep fraction	*r* = 0.688, *r*^2^ = 0.473	*r* = 0.900, *r*^2^ = 0.810	*r* = 0.915, *r*^2^ = 0.837
Deep sleep fraction	*r* = 0.294, *r*^2^ = 0.086	*r* = 0.906, *r*^2^ = 0.816	*r* = 0.964, *r*^2^ = 0.928
REM sleep fraction	*r* = 0.831, *r*^2^ = 0.691	*r* = 0.853, *r*^2^ = 0.727	*r* = 0.865, *r*^2^ = 0.749

### Model interpretability

**Fig. 6:**
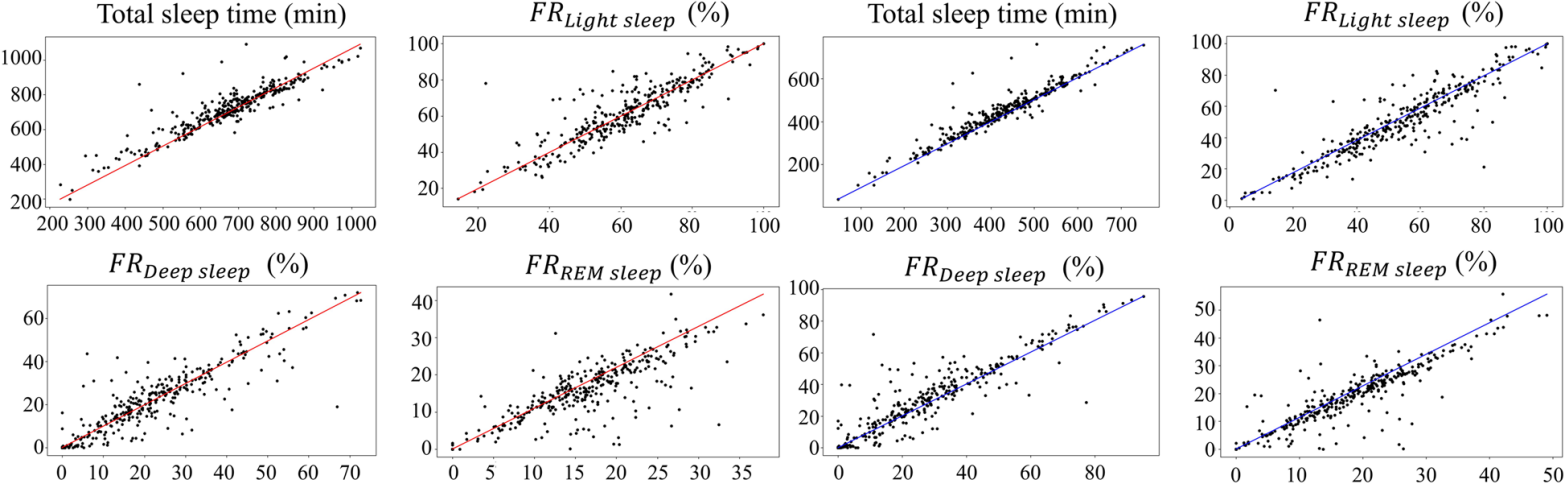
Local attention visualization. The subjects were randomly selected from the CFS dataset

To interpret the rationale behind the model predictions, we conducted an analysis of the local attention module within the model’s architecture. The inclusion of a causal convolutional layer in the local attention module enabled the consideration of sequence relevance across epochs for the entire continuous PPG sequence. Additionally, the final sigmoid function computed important time points for each epoch. The attention, which was computed using the sigmoid function, underwent normalization and subsequent visualization. To visualize the model predictions, we conducted a case study involving three subjects, with one epoch for each class (Fig. 6).

## Discussion

### Overview

In this study, we introduced an algorithm for analyzing sleep stages based on PPG signals. We devised a model with an architecture that is advantageous for time series processing, incorporating InceptionTime, TCN, and an attention module, enabling interpretation of model predictions. We performed selective classification by rejecting ambiguous samples using the energy distribution derived from training set in each dataset. We empirically determined four different energy score thresholds based on the energy distribution and proceeded with our research. As our results demonstrated, employing energy score thresholds led to improved performance across all classes compared to when the energy score thresholds were not applied. When the energy score threshold is lowered, only the samples with greater confidence in the classification results remain, leading to improved performance. However, in real-world applications, it’s important to be cautious about setting the energy score threshold too low, as this can result in the rejection of a substantial amount of data. Therefore, determining the optimal energy score threshold is of utmost importance.

Additionally, we assessed the practicality of applying our method to real-world scenarios using three datasets. We evaluated four sleep parameters (Total Sleep Time, Light fraction, Deep fraction, REM fraction) and found that, even without energy score thresholds, we achieved highly significant correlations for almost all parameters, except deep fraction. Furthermore, applying energy score thresholds improved the correlations compared to the results without thresholds, validating the practical applicability of our approach.

One of the objectives of our study was to enhance the interpretability of the model’s predictions. We achieved this by analyzing the local attention module within the model’s architecture. Upon scrutinizing all three datasets, as depicted in Fig. 6, it became evident that segments exhibiting high attention scores primarily aligned with the peaks of the PPG signal in most of the datasets. This result indicates that the local attention module was focused on the periodicity of the PPG signal, suggesting that our model may leverage physiological characteristics, such as heart rate, respiratory rate, and blood pressure, as previously reported in related studies [11–14], to classify sleep stages.

### Strengths and limitations of the study

In this study, we developed InsightSleepNet, a deep learning-based model designed for automated sleep staging, specifically tailored for continuous PPG data. We integrated a local attention module into the model’s architecture, allowing it to focus more on crucial temporal aspects during training and improving both prediction interpretability and performance. Additionally, we introduced the calculation of energy scores for individual samples, simplifying the assessment of prediction reliability without the need for additional structural complexity. Our objective was not only to evaluate prediction reliability through energy scores but also to enable classification results based on these scores, empowering medical professionals to make more informed judgments. By adopting this approach, we aimed to reduce the need for medical professionals to re-annotate samples with low prediction confidence, potentially easing the workload associated with revising existing annotations. In summary, our model has the capability to enhance the interpretability of model prediction results and estimate uncertainty to reject uncertain predictions. However, several challenges remain. Firstly, the prediction performance for deep sleep stages is not sufficiently high, likely due to class imbalance resulting from a limited number of deep sleep samples. To address this, we plan to employ techniques that consider dataset class ratios to enhance sensitivity toward deep sleep. Additionally, achieving satisfactory performance for the 5-class sleep staging, particularly using PPG data, remains a challenge. To overcome this, we aim to develop a more detailed model that accurately captures the cardiovascular characteristics specific to each sleep stage. Furthermore, while the energy-based uncertainty estimation method has several advantages, determining the optimal threshold still relies on empirical methods. Therefore, our goal is to develop a method based on a more specific theoretical foundation to establish the threshold. Lastly, while our model provides interpretability for prediction results, further research into quantifying this interpretability could offer even better insights. In our future studies, we intend to address these weaknesses and evaluate the suitability of InsightSleepNet for practical use in analyzing PPG data obtained from wearable devices. Our model was developed for sleep staging using data acquired during sleep rather than for real-time application. However, with appropriate modifications to the structure and purpose of the model, it appears feasible to adapt it for real-time application on wearable devices. In conclusion, this research leads us to anticipate enabling effective continuous sleep monitoring in the future. Through this research, we remain optimistic that our algorithm may enable efficient continuous sleep monitoring in the future.

## Conclusion

In this study, we developed InsightSleepNet, a 4-class sleep staging model tailored for continuous PPG data. This model not only provides interpretability to enhance collaboration between humans and AI but also has the ability to reduce uncertainty in predictions through uncertainty estimation. To the best of our knowledge, InsightSleepNet represents the first research effort aimed at improving the performance of automatic sleep staging algorithms by the ability to reject uncertain predictions and offering interpretability of prediction results. The model’s performance was validated using three public datasets, demonstrating its potential for sleep staging using continuous PPG data. Furthermore, our model incorporated a local attention module as part of its architecture, enabling us to analyze which parts of the PPG signal it focuses on when making predictions. This enhances our understanding of the interpretation of prediction results. We also utilized an energy-based uncertainty measure to estimate the confidence level of prediction results, and its performance was verified. InsightSleepNet not only facilitates the continuous sleep monitoring through wearable devices but also supports the evaluation of various factors (such as food intake, fatigue, pharmaceutical effect, daily events) that impact the sleep quality - factors that can be challenging to evaluate through polysomnography. Moreover, the model’s interpretability is paramount in empowering medical professionals to make well-informed decisions. By leveraging energy-based decision-making, our model excels in identifying uncertainties, leading to more precise and reliable sleep quality assessments in collaboration with medical experts. In conclusion, this model expands the role of AI in the medical domain, providing continuous oversight of patients’ sleep health and offering interpretability.

## Data Availability

MESA and CFS datasets are available at the National sleep research resource (NSRR) (https://sleepdata.org/). CAP dataset is available at the physionet.org repository (https://physionet.org/content/challenge-2018/1.0.0/). Our source codes used for this study are available from the GitHub repository. (https://github.com/BorumNam/InsightSleepNet/blob/main/InsightSleepNet.ipynb).

## Abbreviations

AASM: American academy of sleep medicine
REM: Rapid eye movement
EEG: Electroencephalography
EOG: Electrooculography
EMG: Electromyography
ECG: Electrocardiogram
PPG: Photoplethysmography
LSTM: Long short term memory
CNN: Convolutional neural network
GRU: Bidirectional gated recurrent unit
SOTA: State-of-the-art
ResConv: Residual convolution
TCN: Temporal convolutional network
AI: Artificial intelligence
ReLU: Rectified linear unit
OOD: Out-of-distribution
EBM: Energy-based model
MESA: Muti-Ethnic Study of Atherosclerosis
CFS: Cleveland Family study
CAP: Cyclic Alternating Pattern
R&K: Rechtschaffen & Kales
NLL: Negative log likelihood loss
TST: Total sleep time
